# Exploring the Varying Interest in Rural Medicine and Associated Factors Among Medical Students in Japan: A Cross-Sectional Study

**DOI:** 10.7759/cureus.55743

**Published:** 2024-03-07

**Authors:** Asuka Kikuchi, Ryuichi Kawamoto, Daisuke Ninomiya, Yoshio Tokumoto, Teru Kumagi

**Affiliations:** 1 Department of Community Medicine, Ehime University Graduate School of Medicine, Toon, JPN; 2 Department of Community Medicine, Ehime University, Toon, JPN; 3 Postgraduate Medical Education Center, Ehime University Hospital, Toon, JPN

**Keywords:** rural orientation, career aspiration, specialty preference, rural practice, medical school students

## Abstract

Background and objective

Examining the factors influencing the career aspirations of medical students is imperative for understanding their orientation toward rural medicine. Such an investigation can serve as a basis for shaping medical education curricula dedicated to nurturing rural focus. Although previous studies have categorized students based on the presence or absence of orientation toward rural medicine and explored their sociodemographic characteristics, these students may not constitute a homogeneous group; their interests can range from aspiring to establish residence and professional practice in a specific region to being merely willing to endure brief regional placements. There is a scarcity of comprehensive examination of the extent and potential variations of rural orientation in the literature. Our survey addresses this gap by exploring the variations in rural orientation among medical students and the differences in their sociodemographic characteristics and preferred specialties based on their degree of rural orientation.

Methods

We classified medical students into four groups according to their levels of rural orientation: demonstrating proactive engagement towards it, considering it for a defined duration, indicating a preference for avoiding it, and considering it unfeasible. The distribution within each group was investigated. A subsequent analysis of rural orientation and its associated sociodemographic characteristics was performed: a conventional dichotomous study was conducted based on the presence or absence of rural orientation, and a focused study compared students actively interested in rural healthcare with other students. This approach enabled us to explore differences in the degree of rural orientation and associated factors.

Results

The study included 531 students, with 89 participants demonstrating proactive engagement towards rural medicine, 283 considering it for a defined period, 95 indicating an inclination to avoid it, and 63 students stating that it is unfeasible for them. Associated sociodemographic characteristics were explored based on the presence or absence of rural orientation and included recommendations for admission by a designated high school, the presence of a physician role model, and aspirations for obstetrics and gynecology departments. Conversely, when exclusively focusing on students with a desire for proactive engagement in rural medicine, positive correlations were observed with characteristics such as being from the same non-urban prefecture as that of the university where the study was conducted, having a history of residing in a rural area, having a physician role model, and expressing aspirations for general practice or family medicine. Aspiring to be an organ-specific specialist showed a negative correlation with high levels of rural orientation.

Conclusions

Based on our findings, rural orientation is not uniform among medical students; distinct levels of this aspect were observed, each associated with different sociodemographic factors.

## Introduction

The persistent challenge relating to the shortage of physicians in rural areas is a serious concern globally [1,2]. This issue is particularly pronounced in Japan, where the rapidly aging population has exacerbated the problem. Japan’s physician-to-population ratio has fallen below the global average of 3.4 physicians per 1,000 people, and it currently stands at 2.6 physicians per 1,000 people [3]. Among member nations of the Organization for Economic Cooperation and Development (OECD), Japan ranks 29th out of 37 countries in terms of physician density [3]. Furthermore, there is considerable regional disparity in physician distribution in Japan, with rural areas within the same prefecture having only 2/3 to 1/10 of the physicians per 100,000 people in urban centers [4]. These prevailing circumstances have contributed to a chronic shortage of healthcare professionals and presented ongoing challenges.

The Japanese government has implemented various initiatives in response to these challenges [5,6]. Notably, the Jichi Medical University was established in 1972 with a mandatory rural community service requirement to increase the number of physicians in these areas [5]. National efforts to address the shortage since 2008 have included expanding medical school enrollment capacity and introducing a regional quota system at national universities [6]. Despite these changes, imbalance among medical specialties has persisted due to a lack of restrictions on physicians’ career choices; in 2018, the establishment of the Japanese Medical Specialty Board system marked a transition towards a unified management framework overseeing specialized medical practitioners. Under this new system, limitations have been placed on the number of trainee doctors by medical specialty for each prefecture. Still, the same trends have persisted, with urban areas remaining more popular for initial training compared to rural regions [7].

In light of these concerns, medical educators have undertaken empirical investigations into the determinants of rural orientation to identify strategic points of intervention that could foster medical students’ interest in rural communities [8-12]. The identified sociodemographic characteristics associated with rural orientation include having a history of living in a rural community, having general work experience, having a physician role model, and possessing high self-efficacy in rural medicine [8-12]. Aspirations toward general practice or family practice have been associated with high levels of rural orientation as well [13-15]. Most of these studies used a "Yes/No" question to identify students' interest in rural community medicine and reported that students who answered “Yes” had an interest in rural community medicine [8-10,12,13,15].

However, the dichotomous classification underlying these previous approaches has raised pertinent questions. Qualitative and quantitative investigations have reported that medical students expressing a desire to participate in rural healthcare form a diverse group with varying levels of commitment, ranging from those actively seeking a rural community-based lifestyle to those willing to tolerate it for a limited period [11,14,16]. Despite this element of diversity, there has not yet been a comprehensive examination of medical students’ rural orientation, assessing sociodemographic factors or career paths related to different levels of rural orientation. Our study aims to address this gap by exploring the differences in the degree of rural orientation among medical students in Japan, along with variations in their sociodemographic characteristics and preferred specialties.

## Materials and methods

Study design and participants

We conducted a cross-sectional observational study from April 2019 to March 2021 at the Ehime University School of Medicine, a national university in Japan. The target population comprised all medical students enrolled at the university during the study period. Careful attention was paid to prevent duplication of participants across academic years during the survey period. Students were distributed paper-based questionnaires during classes and community practices and were instructed to complete them. For those who did not participate in these sessions, invitations to respond to the survey online were extended through email and social media platforms.

Questionnaire

In this study, a specifically designed survey instrument was utilized due to the absence of prior reports investigating the stages of rural orientation and background factors on a stage-by-stage basis (Appendices). The subsequent sections delineate the methodology involved in creating the survey instrument, covering the definition of terms and referring to relevant prior studies in the formulation of background factors [7,13,14,16-19].

Definition of rural orientation

The definition of rural orientation, informed by previous research, was as follows: when asked about potential future involvement in rural community healthcare, students who affirmed this possibility were labeled as having rural orientation; those who denied it were considered to lack rural orientation [13,14]. To assess the degree of rural orientation, students contemplating rural work were divided into two groups: those expressing a strong desire for active engagement, and those willing to participate for a specified duration. Students unwilling to work rurally were also divided into two categories: those aiming to avoid such engagement as much as possible, and those deeming it unfeasible for themselves. This classification therefore encompassed four levels of rural orientation. Students with a high level of rural orientation were those demonstrating a proactive desire for engagement, while those expressing a desire to participate for a specified duration had a moderate level of rural orientation.

Sociodemographic characteristics

Characteristics examined included the following: sex; location and urbanization of the student’s hometown; type of high school attended; whether they graduated from a combined junior and senior high school; whether they had waited for more than a year for another chance to enter university; whether they had withdrawn from a university to enter medical school; whether they received a scholarship; whether they received a recommendation from a designated high school; and whether they had a physician role model [14,16,17]. Some of the sociodemographic factors examined were tailored to the Japanese higher education system [17,18]. The designated school recommendation system is a system in the Japanese education context where certain high schools are granted the authority to recommend their students for admission to specific universities or programs. This recommendation is often based on the student's academic achievements, extracurricular activities, and other qualifications. It is a unique feature of the Japanese high school education system, providing students with an alternative pathway to university admission.

Specialty preference

The assessment of specialty preference included the basic specialties certified by the Japanese Medical Specialty Board: general practice (including family medicine), surgery, pediatrics, obstetrics and gynecology, psychology, anesthesiology, emergency medicine, dermatology, plastic surgery, ophthalmology, otolaryngology, orthopedics, urology, radiology, brain surgery, pathology, forensic medicine, and laboratory medicine [7,19]. A free-response section was included for students who preferred a career other than those listed.

Statistical analyses

We used SPSS Statistics, version 27 (IBM Japan Ltd., Tokyo, Japan) for statistical analysis. Only completed questionnaires were included. We initially assessed the level of medical students’ rural orientation according to the four designated categories, examining the distribution of each. Next, two definitions were created for "students with rural orientation," and comparisons were made between students with and without rural orientation under each definition (for Analysis A and B). Subsequently, we explored the sociodemographic factors and career determinants related to rural orientation. The details of the definition of rural orientation are described below. 

In the first analysis (Analysis A), participants were divided into two distinct groups, consistent with the methodologies employed in previous studies: medical students with a high and moderate level of rural orientation were collectively categorized as having rural orientation. Conversely, those who preferred to minimize their involvement in rural medicine or deemed it unfeasible were collectively classified as students without rural orientation. After setting up the groups in this manner, we compared both groups and examined the sociodemographic factors associated with rural orientation as well as preferred medical specialties.

In the second analysis (Analysis B), only students demonstrating a high level of rural orientation were considered as having rural orientation, and comparisons were made with students showing a moderate level of rural orientation, those inclined to avoid it if possible, and those deeming it unfeasible. Subsequently, similar to the previous analysis, we investigated factors related to rural orientation and factors related to the desired specialty. 

This analytical approach of first including and then not including students with a moderate level of rural orientation in the group of students with high rural orientation served to identify the items that truly correlated with a high level of rural orientation, as well as to determine the factors that may have influenced moderate rural orientation. An analysis of variance (ANOVA) was conducted to examine the differences in sociodemographic characteristics and specialty preferences between students with and without rural orientation. The dependent variables showing an association with rural preference were identified, and subsequent logistic regression analysis was performed on the variables exhibiting significant differences.

Informed consent and ethical considerations

The purpose of the study was clearly outlined at the outset of the distributed questionnaire. It was explained that answering the questionnaire would constitute consent to cooperation in the study. We obtained approval from the Institutional Review Board at Ehime University Hospital for this study (reference number: 15007004, dated July 27, 2015).

## Results

**Figure 1:**
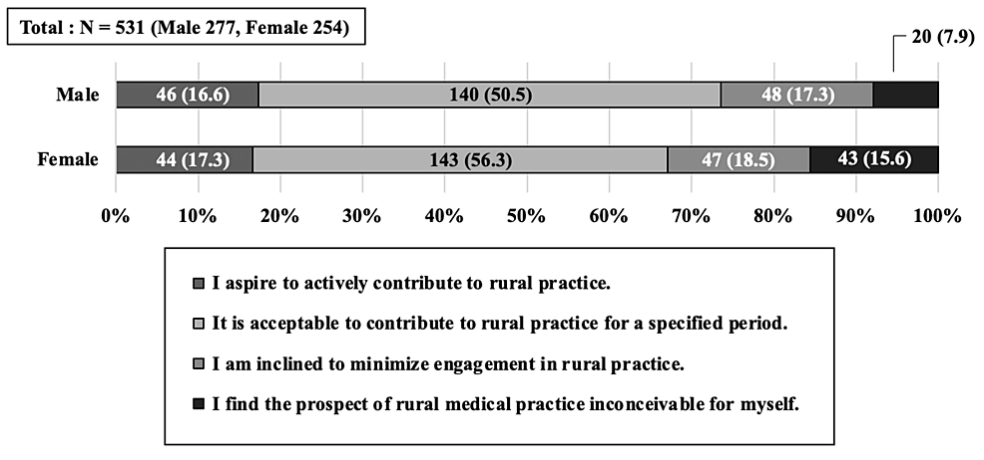
Distribution of students based on the degree of rural orientation Students who chose “I aspire to contribute actively to rural practice” were categorized as students with a high level of rural orientation, and those who responded with “It would be acceptable to contribute to rural practice for a specified period” were categorized as students with a moderate level of rural orientation

A total of 531 out of 697 students completed the questionnaire, resulting in a collection rate of 76.2%, and there were no withdrawals from the study. Of the respondents, 373 medical students (70.1%) expressed a willingness to participate in rural medicine. Within this group, 89 students (16.8%) demonstrated a high level of rural orientation, while 283 students (53.2%) expressed a willingness to engage in rural medicine for a specified period. This distribution indicates a higher prevalence of students with a moderate level of rural orientation compared to those with a higher level; 159 students demonstrated low rural orientation, among whom 95 students indicated a preference to avoid working in the region, and 63 students deemed it inconceivable for them (Figure 1).

Analysis A: students exhibiting high and moderate levels of rural orientation vs. those without rural orientation

Results of the examination of medical students according to their willingness to engage in future rural practice revealed a correlation between several sociodemographic factors with rural orientation, including being from the same non-urban prefecture as that of the university where the study was conducted, having general work experience, receiving a recommendation for admission by a designated high school, obtaining a scholarship, and having a physician role model. Furthermore, an inclination toward obstetrics and gynecology as a desired future specialty exhibited a significant association with rural orientation.

Analysis B: students demonstrating a high rural orientation vs. all other students

The findings indicated that the factors associated with a high level of rural orientation included being from the same non-urban prefecture as that of the university where the study was conducted, having a history of residing in a rural area, and having a physician role model (Table 1). In terms of desired medical specialties, aspiring to be a general practitioner or family physician, not aspiring to be an organ-specific specialist, and not aspiring to be an orthopedic surgeon were found to be relevant factors (Table 2).

**Table 1:** Differences in sociodemographic characteristics between medical students with or without rural orientation Table 1 shows the sociodemographic characteristics of students with and without intention for rural practice (rural orientation). In Analysis A, students with rural orientation included students with a strong desire for active engagement and students willing to participate for a specified duration. In each analysis, we compared students with rural orientation to those without rural orientation, assessed statistical significance using the χ2-test for categorical variables, and represented the results with p-values. Significant values (p<0.05) are presented in bold

Sociodemographic characteristics	N/A	Rural orientation					
		Analysis A			Analysis B		
		Students with a strong desire for active engagement and students willing to participate for a specified duration, n (%)			Students with a strong desire for active engagement, n (%)		
N/A	N/A	Yes (n = 373)	No (n = 158)	P-value	Yes (n = 90)	No (n = 442)	P-value
Sex	Male	186 (49.9)	91 (57.6)	0.103	44 (48.9)	231 (52.4)	0.826
	Female	187 (50.1)	67 (42.4)		46 (51.1)	210 (47.6)	
Being from the same non-urban prefecture as that of the university where the study was conducted	N/A	196 (52.5)	53 (33.5)	<0.001	55 (61.1)	194 (44.0)	0.003
Type of high school	Public	174 (46.6)	74 (46.8)	0.969	43 (47.8)	205 (46.5)	0.823
	Non-public: private, national, other	199 (53.4)	84 (53.2)		47 (52.2)	236 (53.5)	
Graduated from combined junior and senior high school	N/A	214 (57.4)	81 (51.3)	0.195	49 (54.4)	246 (55.8)	0.816
Having waited more than a year for another chance to enter university	N/A	143 (38.3)	70 (44.3)	0.200	36 (40.0)	177 (40.1)	0.981
Having general work experience	N/A	11 (2.9)	10 (6.3)	0.068	4 (4.4)	17 (3.9)	0.794
Having withdrawn from a university and entered medical school	N/A	23 (6.2)	18 (11.4)	0.039	6 (6.7)	35 (7.9)	0.681
Having received scholarship	N/A	132 (35.4)	39 (24.7)	0.016	36 (40.0)	135 (30.6)	0.082
Having received a recommendation from a designated high school	N/A	141 (37.8)	27 (17.1)	<0.001	34 (37.8)	134 (30.4)	0.169
Having been raised in a rural area	N/A	21 (5.6)	9 (5.7)	0.976	13 (14.4)	17 (3.9)	<0.001
Having a physician role model	N/A	175 (46.9)	44 (27.8)	<0.001	53 (58.9)	166 (37.6)	<0.001

**Table 2:** Differences in specialty preference between medical students with and without rural orientation Table 2 shows the differences in specialty preference between students with and without intention for rural practice (rural orientation). In Analysis A, students with rural orientation included students with a strong desire for active engagement and students willing to participate for a specified duration. In Analysis B, students with rural orientation only included students with a strong desire for active engagement. In each analysis, we compared students with rural orientation to those without rural orientation, assessed statistical significance using the χ2-test for categorical variables, and represented the results with p-values. Significant values (p<0.05) are presented in bold

Specialty preference	Rural orientation					
	Analysis A			Analysis B		
	Students with a strong desire for active engagement and students willing to participate for a specified duration, n (%)			Students with a strong desire for active engagement, n (%)		
N/A	Yes (n = 372)	No (n = 159)	P-value	Yes (n = 89)	No (n = 442)	P-value
General practice/family medicine	207 (55.5)	75 (47.5)	0.090	62 (68.9)	220 (49.9)	0.001
Internists with subspecialties	102 (27.3)	39 (24.7)	0.525	15 (16.7)	126 (28.6)	0.020
Surgery	115 (30.8)	49 (31.0)	0.967	31 (34.4)	133 (30.2)	0.423
Pediatrics	106 (28.4)	43 (27.2)	0.778	28 (31.1)	121 (27.4)	0.480
Obstetrics and gynecology	83 (22.3)	22 (13.9)	0.028	19 (21.1)	86 (19.5)	0.727
Psychology	62 (16.6)	30 (19.0)	0.510	15 (16.7)	75 (17.5)	0.856
Anesthesiology	80 (21.4)	34 (21.5)	0.985	17 (18.9)	97 (22.0)	0.513
Emergency medicine	82 (22.0)	25 (15.8)	0.106	24 (26.7)	83 (18.8)	0.091
Dermatology	43 (11.6)	15 (9.4)	0.472	5 (5.6)	53 (12.0)	0.073
Plastic surgery	2 (0.5)	1 (0.6)	0.892	0 (0.0)	3 (0.7)	0.433
Ophthalmology	41 (11.0)	16 (10.1)	0.768	9 (10.0)	48 (10.9)	0.805
Otolaryngology	34 (9.1)	10 (6.3)	0.287	5 (5.6)	39 (8.8)	0.302
Orthopedics	57 (15.3)	27 (17.1)	0.602	7 (7.8)	77 (17.5)	0.022
Urology	18 (4.8)	10 (6.3)	0.479	2 (2.2)	26 (5.9)	0.155
Radiology	50 (13.4)	19 (12.0)	0.666	8 (8.9)	61 (13.8)	0.204
Brain surgery	1 (0.3)	2 (1.3)	0.161	0 (0.0)	3 (0.7)	0.433
Pathology	3 (0.8)	1 (0.6)	0.835	1 (1.1)	3 (0.7)	0.667
Forensic medicine	1 (0.3)	0 (0.0)	0.515	0 (0.0)	1 (0.2)	0.651
Laboratory medicine	0 (0.0)	0 (0.0)	-	0 (0.0)	0 (0.0)	-
Others	4 (1.1)	1 (0.6)	0.632	0 (0.0)	5 (1.1)	0.310

Multivariate analysis

A binomial logistic regression analysis was performed, by selecting items associated with rural orientation from Analyses A and B. Outcomes revealed that, in Analysis A, the factors ultimately linked to rural orientation included being recommended for admission by a designated high school, having a physician role model, and aspiring to work in obstetrics and gynecology departments. In Analysis B, factors such as being from the same non-urban prefecture as that of the university where the study was conducted, having a history of residing in a rural area, having a physician role model, and expressing aspirations for general practice or family medicine were positively associated with rural orientation. Notably, not aspiring to be an organ-specific specialist was linked with a high level of rural orientation (Table 3).

**Table 3:** Odds ratios (ORs) of variables related to students with rural orientation Table 3 shows ORs of variables related to students with an intention for rural practice (rural orientation). In Analysis A, students with rural orientation included students with a strong desire for active engagement and students willing to participate for a specified duration. In Analysis B, students with rural orientation only included students with a strong desire for active engagement. Variables showing significance in ANOVA were analyzed. Variables associated with the students without clear rural orientation are shown in bold

Variables related to students with rural orientation	Rural orientation							
	Analysis A				Analysis B			
	Students with a strong desire for active engagement and students willing to participate for a specified duration (yes, n = 372; no, n = 159)				Students with a strong desire for active engagement (yes, n = 89; no, n = 442)			
	Stepwise regression		Forced regression		Stepwise regression		Forced regression	
	OR (95% CI)	P-value	OR (95% CI)	P-value	OR (95% CI)	P-value	OR (95% CI)	P-value
Sociodemographic characteristics								
Being from the same non-urban prefecture as that of the university where the study was conducted	1.69 (1.10–2.60)	0.017	1.55 (0.99–2.40)	0.052	1.79 (1.10–2.91)	0.019	1.79 (1.10–2.91)	0.019
Having withdrawn from a university and entered medical school	0.55 (0.28–1.09)	0.086	0.62 (0.32–1.23)	0.174	--	--	--	--
Having received a scholarship	2.09 (1.26–3.47)	0.004	1.39 (0.89–2.17)	0.153	--	--	--	--
Having received a recommendation from a designated high school	2.31 (1.39–3.84)	0.001	2.19 (1.31–3.65)	0.003	--	--	--	--
Having been raised in a rural area	--	--	--	--	3.39 (1.50–7.65)	0.019	3.39 (1.50–7.65)	0.019
Having a physician role model	2.27 (1.50–3.44)	<0.001	2.33 (1.54–3.55)	<0.001	2.08 (1.28–3.36)	0.003	2.08 (1.28–3.36)	0.003
Specialty preference								
General practice/family medicine	--	--	--	--	2.02 (1.21–3.35)	0.007	2.02 (1.21–3.35)	0.007
Internists with subspecialties	--	--	--	--	0.48 (0.26–0.89)	0.019	0.48 (0.26–0.89)	0.019
Obstetrics and gynecology	1.80 (1.06–3.06)	0.030	1.87 (1.10–3.18)	0.021	--	--	--	--
Orthopedics	--	--	--	--	0.44 (0.19–1.04)	0.060	0.44 (0.19–1.04)	0.060

## Discussion

This study revealed several nuances regarding types of rural orientation among medical students, indicating that a notable proportion expressed willingness to engage in rural practice for a limited period rather than a prolonged involvement. Factors positively associated with high rural orientation included being from the same prefecture as the university of interest, having a history of residing in rural areas, having a physician role model, and expressing an interest in general practice or family medicine specialties. Conversely, aspiring to become an organ-specific specialist demonstrated a negative correlation with rural orientation.

Variations in rural orientation

This investigation highlighted the heterogeneous nature of medical students’ rural orientation, with some actively seeking engagement in rural medicine and others willing to do so only for a defined period. Notably, the latter inclination was more prevalent. To our knowledge, no prior studies have systematically delineated distinct levels of rural orientation. In semi-structured interviews with medical students expressing an interest in rural medicine, Tolhurst et al. [16] observed instances where rural medicine was perceived as a short-term adventure. Additionally, in a survey involving medical students obliged to practice in rural communities post-graduation for financial support, Sempowski et al. [20] reported initial success in recruitment but challenges in sustaining physician numbers in these communities long-term. These findings suggest that medical students might view rural community practice as a temporary and conditional prospect rather than a lifelong commitment.

Our identification of the levels of rural orientation therefore aligns with and contributes to existing literature. Efforts to foster rural orientation should take into account these stages of interest. For example, students with an already high level of rural orientation may benefit from practical training experiences in rural communities [10,21]. However, for those with limited rural orientation, a flexible system allowing engagement for a defined period according to each individual’s career plans may be a more strategic approach to cultivating a lasting commitment to rural practice in the future [22].

Degree of rural orientation, sociodemographic characteristics, and preferred medical specialties

Our findings showed that the sociodemographic factors and career paths associated with rural orientation varied depending on the degree of rural orientation. Factors such as being born in the same prefecture as the university of interest and having a rural upbringing were linked to a higher degree of rural orientation. This aligns with existing reports indicating a correlation between a strong affinity for a rural area and an increased level of rural orientation [6,7,9,20]. The results from the examination of students’ preferred medical specialties are also consistent with prior findings, emphasizing that students recognize the importance of rural healthcare; students interested in general practice and family medicine, in particular, tend to favor rural medicine [13-15]. Notably, some medical students in this study expressed an intention to accept rural employment for a limited period, even without prior residency in the area.

Another significant point was that obtaining an organ-specific specialist education in internal medicine was challenging within rural regions, which may cause hesitation and act as a barrier for students with limited rural orientation. Efforts to alleviate physician shortages in rural areas included financial assistance, obligatory rural practice policies, and rural training programs. However, the long-term impact of these initiatives remained uncertain [5,20]. Factors such as low self-efficacy and interpersonal issues have been identified as reasons why physicians avoid prolonged stays in rural areas, which present potential obstacles to the sustained effectiveness of existing policies [12,23]. Moreover, given that many medical students exhibit limited inclinations toward rural medicine, it may be necessary to explore alternative frameworks for effectively encouraging and supporting these students in taking up rural healthcare. This strategy is distinct from efforts aimed at cultivating high levels of rural orientation among students.

One strategy for addressing limitations in rural healthcare is an initiative known as specialist outreach. By deploying specialists to areas with limited healthcare access, the referral rate to specialists has been reported to increase, and patients’ burden of seeking medical care in urban facilities is alleviated [24]. Furthermore, reports have suggested that outreach practiced in collaboration with regional healthcare systems leads to more efficient and guideline-compliant care, which contributes to improved health outcomes [25]. This highlights a way through which specialists can contribute to rural healthcare while also working in urban areas; this can also help achieve the short-term involvement of doctors in rural healthcare and relieve barriers that people in rural areas may face in obtaining organ-specific specialty care. Training physicians to contribute actively on the front lines of rural healthcare is an important future challenge. An assessment of the rural orientation and career development trends of medical students in the millennial generation indicates that there may be room to strengthen rural healthcare with the help of physicians who do not permanently reside in the region.

Limitations

The respondents in this study were predominantly Japanese medical students, and we specifically targeted students aspiring to become physicians through the Japanese medical education system. The selection of future medical specialties was also confined to the prefectural level in Japan, where such determinations are limited. Hence, caution is necessary when attempting to generalize the present findings to medical students in other international contexts. Furthermore, this study did not verify medical students’ individual perceptions of rural medicine. Depending on the hometown and upbringing of medical students, the population size and degree of urbanization associated with their desired region may also vary. The potential impact of these considerations on the survey results cannot be ruled out.

The survey instrument utilized in this study was tailored to address the absence of prior research on the strengths and stages of rural orientation. While questions assessing the stages of rural orientation were systematically crafted, no provisions were included for students with an uncertain interest in rural medical practice. Moreover, the phrasing of each option and the specific number of stages for rural orientation were distinctly determined. Consequently, the survey instrument's detection power and reproducibility require further examination in future research. It is essential to bear in mind the potential impacts of these research limitations on the outcomes and the results should be interpreted accordingly.

## Conclusions

This study demonstrated varying levels of rural orientation among medical students and showed a significant preference for short-term engagement in rural medicine over prolonged involvement. High rural orientation correlated positively with factors such as being from the prefecture of the university of interest, having a history of residing in rural areas, having a physician role model, and expressing interest in general practice or family medicine. Conversely, aspiring to become a specific organ specialist exhibited a negative correlation with rural orientation. Further studies should be conducted to verify the validity and reliability of the various types of rural orientation and the explanatory factors for the variation in rural orientation levels as described in this study. Ultimately, we recommend the development of a medical education curriculum that aligns with the career plans of medical students while simultaneously enhancing their rural orientation.

## Appendices

**Table 4:** A survey of background factors and career determinants influencing the rural orientation of medical students The questions without predefined choices are designated for free-form responses. Additionally, respondents are afforded the option to select multiple desired medical specialties for the future

Questionnaire	
Q1. Would you mind sharing your sex with me?	
Q2. May I ask how old you are?	
Q3. What grade are you in?	
Q4. Where are you from?	
Q5. Which type of high school did you attend?	1. Public. 2. Private. 3. National. 4. Others
Q6. Was your high school a combined junior and senior high school?	1. Yes. 2. No
Q7. Have you waited more than a year for another chance to enter a university?	1. Yes. 2. No
Q8. Have you ever participated in a work experience program?	1. Yes. 2. No
Q9. Did you drop out of a university and enroll in medical school?	1. Yes. 2. No
Q10. Do you have a physician role model?	1. Yes. 2. No
Q11. Do you receive funding for your studies from a local government or organization?	1. Yes. 2. No
Q12. Did you enter a medical school with a recommendation?	1. Yes. 2. No
Q13. What is the urban population of the area where you have lived the longest in your life?	1. Large city (over 500,000). 2. Medium-sized city (100,000 to 500,000). 3. Small city (approximately 50,000). 4. Town/village. 5. Remote area/island
Q14. How do you envision your future involvement in rural practice? Please choose the option that resonates with you the most	1. I aspire to actively contribute to rural practice
	2. It is acceptable to contribute to rural practice for a specified period
	3. I am inclined to minimize engagement in rural practice
	4. I find the prospect of rural medical practice inconceivable for myself
Q15. Please select the medical specialties you aspire to pursue in your future career (multiple choices allowed)	
1. General practice (including family medicine). 2. Surgery. 3. Pediatrics. 4. Obstetrics and gynecology. 5. Psychology. 6. Anesthesiology. 7. Emergency medicine. 8. Dermatology. 9. Plastic surgery. 10. Ophthalmology. 11. Otolaryngology. 12. Orthopedics. 13. Urology. 14. Radiology. 15. Brain surgery. 16. Pathology. 17. Forensic medicine. 18. Laboratory medicine. 19. others ( )	
